# Non-Titanised Compared to Titanised Polypropylene Meshes Induce a Pro-Inflammatory and Anti-Angiogenic Response in Fibroblast Co-Cultures

**DOI:** 10.1007/s12013-026-02062-8

**Published:** 2026-04-07

**Authors:** Oleksii Protsepko, Philipp Voisard, Nina Ditsch, Melitta Beatrice Köpke, Christina Kuhn, Birgit Urban, Nicole Pochert, Christian Dannecker, Udo Jeschke, Friedrich Pauli, Fabian Garrido, Carl Mathis Wild

**Affiliations:** 1 Gynecology and Obstetrics, Faculty of Medicine, University of Augsburg, Stenglinstraße 2, Augsburg, Germany; 2 Gynecology, Obstetrics and Senology, Faculty of Medicine, University of Augsburg, Stenglinstraße 2, Augsburg, Germany; 3 Bavarian Centre for Cancer Research (BKFZ), Partner Site Augsburg, Augsburg, Germany; 4 Institute of Environmental Medicine, Helmholtz Center Munich, German Research Center for Environmental Health, Neuherberg, Germany

**Keywords:** Fibroblasts, Mesh surgery, Polypropylene mesh, Titanised polypropylene mesh, sFlt-1, IL-4, IL-16

## Abstract

Pelvic organs prolapse (POP) can be treated surgically with native tissue or by placing mesh implants in the pelvic floor and vaginal wall. Mesh implants are manufactured from different materials. We previously demonstrated that titanised polypropylene meshes induced different macrophage polarization compared to pure polypropylene meshes. However, these differences were only observed in cell cultures where the meshes were encapsulated by human dermal fibroblasts (NHDF). In this study, we co-cultivated titanised and non-titanised polypropylene meshes with NHDF alone to analyze the immune response of NHDF against different mesh materials. Concentration of proteins in cell culture supernatants were analyzed using multiplex ELISA. We identified a significant increase of VEGF and VEGF-C (*p* = 0.001) for both meshes, and the polypropylene meshes induced significant (*p* < 0.001) upregulation of sFlt-1, a vascularization inhibitor. Titanised meshes significantly downregulated (*p* = 0.034) the pro-inflammatory cytokine IL-16, whereas polypropylene meshes showed no effect on IL-16. Polypropylene meshes significantly reduced (*p* = 0.011) the anti-inflammatory cytokine IL-4, while titanised meshes had no effect on IL-4. Polypropylene meshes induce an anti-angiogenic (sFlt-1 upregulation) and pro-inflammatory response. Titanised coating of polypropylene implants reduced proinflammatory cytokines and promoted angiogenesis. These findings have important clinical implications for the postoperative period where fibroblasts play a key role.

## Introduction

Pelvic organ prolapse (POP) represents the protrusion of one or more vaginal walls (anterior or posterior), the uterus (cervix) or the vaginal apex (including the vaginal vault or hysterectomy cuff scar). While POP can remain asymptomatic in some cases, it frequently impacts a woman’s quality of life [1], particularly for older women [2]. Multiple risk factors contribute to POP development, including obesity, genetic predisposition and menopause [3–7]. However, the history of gynaecological and obstetric events represents the most significant risk factor [8,9]. Notably, parity demonstrates the strongest association with POP, where when compared to nulliparous women, those with one child exhibit a fourfold increased risk, while those with two children have an 8.4-fold greater probability of experiencing POP [8].

Currently, prolapse management encompasses both, conservative and operative approaches. Conservative management includes pelvic floor muscle training (PFMT) and mechanical devices such as vaginal pessaries[10,11]. Although PFMT positively impacts prolapse symptoms and severity, research suggests it may be more effective as a preventive measure [12,13]. Nevertheless, conservative approaches offer the notable advantage of fewer adverse effects and complications compared to surgical interventions [14].

When conservative measures fail, affected women require surgical treatment options with low recurrence and complication rates [15]. Surgical methods vary in approach (abdominal or vaginal), including different techniques, fixation points, and the decision of mesh utilization or native tissue alone to repair damaged anatomical structures [16]. At the University Hospital of Augsburg, the refined and optimized titanised polypropylene mesh TiLOOP® PRO A has demonstrated a significant improvement in quality-of-life following implantation for surgical correction of cystocele [17]. Previously, the PROLIFT™ system was also extensively utilised in our clinic [17]. The PROLIFT™ mesh contains nonabsorbable monofilament of polypropylene without titanium coating. Patients with titanium-coated polypropylene (pfmmedical, titanised by PACVD process) mesh reported improved foreign body sensation [18] Systematic reviews indicate that reducing the material load of titanised mesh from 35 to 16 g/m² further improves biocompatibility [19]. In pelvic organ prolapse surgery specifically, laparoscopic sacrocolpopexy with light titanium-coated polypropylene mesh demonstrated low complication rates [20].

Initially, mesh implant use was associated with various complications and adverse outcomes, including erosion, implant failure with accompanied pain, dyspareunia, and delayed-onset infections [21]. Therefore, recent animal studies with modified polypropylene meshes were performed to reduce complication rates [22]. The authors demonstrated that nanofibrous membrane-coated PP mesh effectively reduced the surgical implantation complications and improved the compatibility of PP mesh [22]. Another animal study utilised novel bacterial cellulose (BC) mesh to reduce complications in pelvic floor reconstruction [23]. However, the authors concluded that BC mesh could not represent a promising biomaterial [23]. Very recently, a new rat model was developed to mimic sacrocolpopexy for POP treatment and biomaterials testing [24]. The authors used pure polypropylene (PP) meshes and PP mesh collagen complexes. No significant differences were found between these two mesh complexes [24].

Recent research has demonstrated that the adaptive immune system plays a regulatory role in the foreign body response to mesh implants, with regulatory T cells (Tregs) influencing macrophage polarization and inhibiting CD8+ effector T cell activity [25]. Additionally, in our previous studies we demonstrated that macrophage and cancer cell derived CCL22 can attract FoxP3-positive Treg cells [26,27]. Macrophages are key players in orchestrating local immune responses and tissue reactions [17,28,29]. Our recent study demonstrated that macrophage polarization positive for CD68 and CD163 and expression of immune checkpoint molecules PD-1 and PD-L1 were significantly up- or down-regulated only in inserts where pure polypropylene meshes and titanised polypropylene meshes were co-cultured not only with monocytes but also with fibroblasts [17]. Therefore, we hypothesised that mesh colonization by fibroblasts is necessary to induce different immune responses, according to our recent in vitro approach [17].

CD68- and CD163-positive macrophages play a key role in inducing immunologic tolerance [30,31]. The haemoglobin-haptoglobin complex induces immunosuppressive function of macrophages by activating the CD163 − HO-1 axis [32] and CD163-positive macrophages in the tumour stroma are associated with worse clinical course [33]. Simultaneously, PD-1 and PD-L1 proteins function as immune checkpoint inhibitors, regulating the immune system by providing immune tolerance and downregulating T cell proliferation [34,35]. M1 classically activated macrophages secrete pro-inflammatory cytokines and chemokines like IL-16 and express the general marker CD68 [36]. M2 alternatively activated macrophages associated with anti-inflammatory effect due to IL-4 cytokine and CD163 marker activation [36]. In addition, essential growth factors such as epidermal growth factor (EGF) and vascularisation marker such as VEGF are expressed in normal human dermal fibroblasts (NHDF) [37,38], therefore these cells could act as a fibroblast model in our research approach.

Extensive research has demonstrated that fibroblast-mesh interactions are crucial for biocompatibility assessment. Previous studies have shown that surface-modified polypropylene meshes provide better adhesion, growth, metabolic activity, proliferation, and viability of fibroblasts compared to pure polypropylene meshes alone [39]. Additionally, polypropylene mesh scaffold with adipose-derived stem cells exhibit excellent cellular compatibility, with enhanced cellular adhesion and viability of fibroblast cells, demonstrating reduced inflammatory responses and improved biocompatibility [40].

Based on these data and the established importance of fibroblast-mesh interactions for biocompatibility, we performed in vitro experiments to examine protein and cytokine expression for each mesh co-cultured with normal human dermal fibroblasts (NHDF) and compared the cytokine/vascularisation marker response.

## Materials and Methods

### Fibroblast Cultivation

For cultivation of fibroblasts with two different mesh materials and subsequent determination of proteins in the cell culture supernatants we used the cell line “NHDF” (Promocell, Heidelberg, Germany, adult donor, cryopreserved). According to the supplier information, the cells are pooled and isolated from adult skin from different locations (face, breast, abdomen, and thighs). For the experiments, one vial was obtained from Promocell and multiplied. Twenty thousand NHDF cells (4th passage), in 500 µl medium were seeded in each well of a 24-well-plate, using DMEM (PAN-Biotech, Germany) with 10% FCS (Bio&Sell, Germany) and 1% penicillin-streptomycin-amphotericin (PAN-Biotech, Germany) as cell culture medium.

For the experiments two different mesh materials were used: titanised polypropylene mesh (pfmmedical, titanised by PACVD process; 35 g/m², 1 mm pore size) and polypropylene mesh without titanium coating (pfmmedical; 35 g/m², 1 mm pore size).

**Fig. 1:**
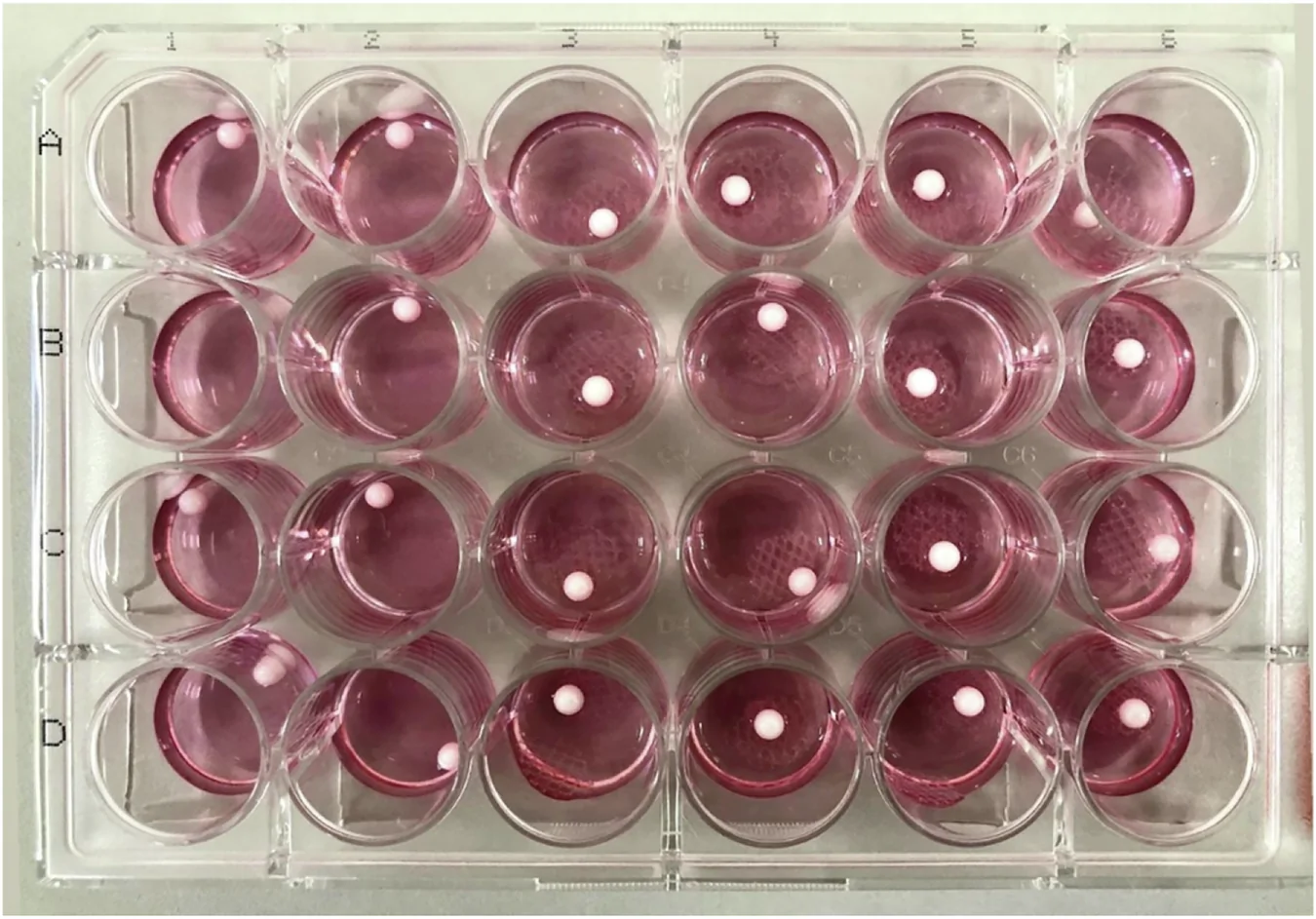
Samples of each culture during co-cultivation in a 24-Well plate: A1-D2 fibroblasts with ceramic beads (white balls), A3-D4 fibroblasts and ceramic beads with titanised meshes, A5-D6 fibroblasts and ceramic beads with polypropylene meshes. Mesh material in columns 3–6 is clearly visible under the ceramic beads

The meshes were laser cut under sterile conditions to a diameter of 5 mm. Eight independent experiments were conducted for each experimental group with two samples measured within each group (Fig. 1). In total, 16 measurements were included for the analysis. One independent experiment corresponds to a complete biological experimental run performed under identical conditions. For each experiment, supernatant samples were collected from two spatially distinct locations within each well, as a homogeneous distribution of analytes across the well cannot be assumed. Consequently, these samples were considered independent observations rather than technical replicates, and each was included as a separate data point in the analysis (*n* = 16 per group; 8 experiments × 2 spatially distinct samples). NHDF with a ceramic bead served as the control group, whilst the subsequent wells contained additionally two cuts of titanised mesh or polypropylene mesh respectively. The ceramic beads were used to anchor the meshes to the bottom of the wells and prevent them from floating at the surface. A control group consisting of NHDFs alone was not included, as ceramic beads may also influence fibroblast behaviour, and the experimental design was intentionally kept as simple as possible. In our initial publication, monocytes were co-cultured with fibroblasts and meshes for 5 days, as macrophages began to die beyond this time point. To maintain consistency in experimental setup and conditions, fibroblasts in the present study were also incubated with meshes for 5 days at 37 °C and 5% CO_2_. Subsequently, cell culture supernatants were collected and stored at −80 °C for further examinations.

### Protein Determination

For measurement of cytokines, chemokines and proteins in the collected cell culture supernatants (one measurement for each vial), commercial multi-array immunoassays were used.

#### ELISA Description

U-Plex Biomarker Group1 (IL-4, IL-16) (MSD; Nr.K15067M-1;Lot: 488032), and Angiogenesis Panel 1 V-PLEX assay platform (Meso Scale Discovery, Rockville, MD), based on electrochemiluminescence detection of Angiogenesis Panel 1_V-Plex Custom Biomarkers (VEGF, VEGF-C, Flt-1, bFGF) - MSD; Nr. K151A9H-1 Lot: 470233; A151A6H-1; Lot: K0082250)) containing multi-array 96-well plates.

#### ELISA Validation

Each assay within the panel was individually validated for specificity by testing single calibrators alongside individual detection antibodies. Non-specific binding remained below 0.5% across all assays. The multi-array plates were pre-coated with capture antibodies targeting the proteins of interest, immobilized on distinct and well-defined spots.

#### Sample Preparation

For the angiogenesis panel, the well-plate was initially treated with a blocking solution for 1 h. After a washing step in washing buffer, appropriate calibrators and cell culture supernatants were added to the well-plates and incubated for 2 h for the angiogenesis panel or 1 h for biomarker group 1. Both plates were washed subsequently. The respective detection-antibody solutions conjugated with electro chemiluminescent labels were then applied for the same incubation times as described for the calibrators and supernatants, followed by an additional washing step. Finally, read buffers were added and the plates were read in the MSD instrument (MESO QuickPlex SQ 120MM). During this process, voltage was applied to the plate electrodes, inducing the captured labels to emit light. The intensity of this emitted signal served as a quantitative indicator of the target protein concentration within the sample [41,42].

#### Calibration and Concentration Determination

The calibration curves used to calculate analyte concentrations were established by fitting the signals from the calibrators to a 4-parameter logistic (or sigmoidal dose-response) model with a 1/y² weighting. The weighting function provides a better fitting of data over a wide dynamic range, particularly at the low end of the calibration curve. Analyte concentrations were determined from the ECL signals by back-fitting to the calibration curve. These assays possess a wide dynamic range (4 logs), which allows accurate quantification of samples without requiring multiple dilutions or repeated testing. The calculations to establish calibration curves and determine concentrations were performed using the MSD DISCOVERY WORKBENCH® analysis software. Optimal quantification of unknown samples was achieved by generating a calibration curve for each plate using a minimum of two replicates at each calibrator level. Median Low Limit of Detection and Detection Range for each analyte tested is shown in Suppl. Table 1. Intra Assay and Intra Lot Variation for each analyte tested is shown in Suppl. Table 2. (see homepage: www.mesoscale.com) [43].

### Statistical Analysis

The statistical analysis was performed by the SPSS 26.0 program (IBM Corp., Armonk, NY, USA). The results obtained were recorded and entered the SPSS database. Due to the small sample size and the characteristics of biological cell culture data, a non-parametric approach was chosen. Wilcoxon signed-rank test for related samples was used, as these test does not require normality assumptions and is more robust for biological data with potentially heterogeneous cellular responses. The significance level was maintained at *p* = 0.05 to ensure adequate statistical power given the small sample size.

We evaluated the association between cytokine expression and vascular factor expression in between matched groups (titanised mesh/polypropylene mesh/control group). Violin plots of 16 measurements in eight individual experiments contain the median and distribution, representing the expression of concentrations in height and frequency of each concentration by chart width. To compare controls and mesh co-cultured fibroblasts a Wilcoxon test was used.

## Results

Normal human dermal fibroblasts (NHDF) were co-cultivated in a 24-well plate for 5 days with inserts containing fibroblasts with ceramic beads (used as a control), polypropylene meshes covered with fibroblasts under the ceramic beads, and titanised meshes covered with fibroblasts under the ceramic beads. Subsequently, the concentration of cytokines, chemokines and proteins in the collected cell culture supernatants from the eight independent experiments were measured. For each experimental group values were measured in two samples using commercial multi-array immunoassays (U-Plex Biomarker Group1 and Angiogenesis Panel 1), containing multi-array 96-well plates.

**Fig. 2:**
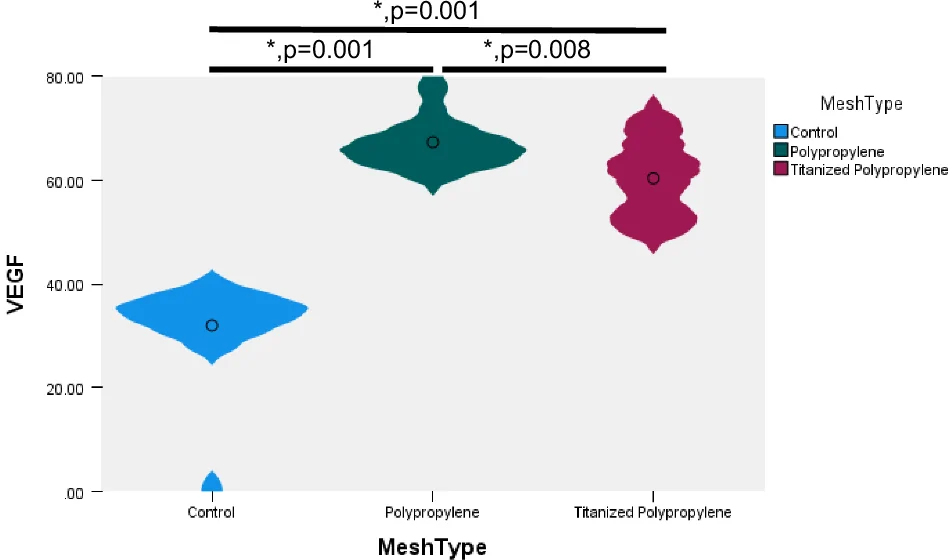
Both cell cultures of polypropylene and titanised meshes covered with fibroblasts under ceramic beads, presenting significantly increased concentration of VEGF from Median = 34.04 pg/ml to Median = 65.64 pg/ml for polypropylene meshes (*p* = 0.001), and Median = 34.04 pg/ml to Median = 61.22 pg/ml for titanised meshes(marked with an asterisk; *p* = 0.001), in comparison to controls in 16 measurements (8 independent experiments measured in two samples)

**VEGF** concentration significantly increased from 34.04 pg/ml (median) in controls with only fibroblasts under the ceramic beads, to 65.64 pg/ml (median) in cell cultures of polypropylene meshes covered with fibroblasts under ceramic beads (*p* = 0.001) and to 61.22 pg/ml (median) in cell cultures of titanised meshes covered with fibroblasts under ceramic beads (*p* = 0.001). In addition, fibroblasts cultured with polypropylene meshes compared to fibroblasts cultured with titanised meshes showed significant differences (*p* = 0.008) (Fig. 2).

**Fig. 3:**
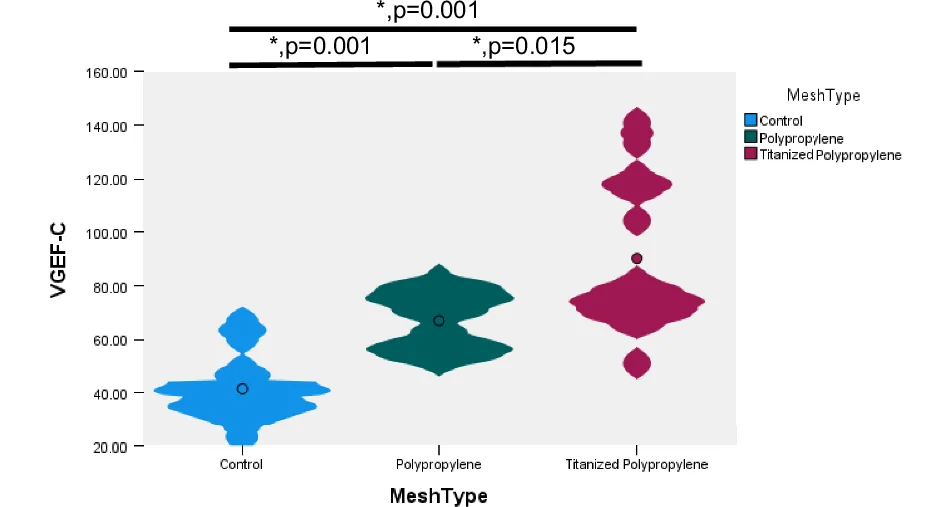
The concentration of the VEGF-C angiogenesis factor significantly increased from Median = 38.91 pg/ml to Median = 69.47 pg/ml in cell cultures of polypropylene meshes covered with fibroblasts under ceramic beads (*p* = 0.001), as well as in cell cultures of titanised meshes covered with fibroblasts under ceramic beads from Median = 38.91 pg/ml in controls to Median = 77.24 pg/ml(*p* = 0.001) in comparison to controls in 16 measurements (8 independent experiments measured in two samples)

**VEGF-C** concentrations significantly increased from median = 38.91 pg/ml in controls with only fibroblasts under ceramic beads, to median = 69.47 pg/ml in cell cultures of polypropylene meshes (*p* = 0.001) and to median = 77.24 pg/ml in inserts with titanised meshes covered with fibroblasts under ceramic beads (*p* = 0.001). In addition, also fibroblasts cultured with polypropylene meshes compared to fibroblasts cultured with titanised meshes showed significant differences (*p* = 0.015) (Fig. 3).

**Fig. 4:**
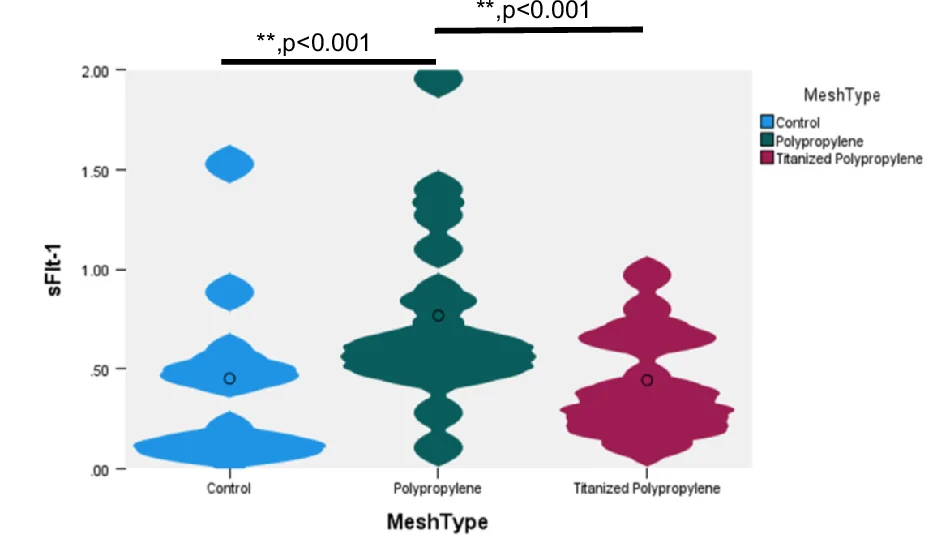
Cell cultures of polypropylene mesh covered with fibroblasts under ceramic beads, presenting increased concentration of anti-angiogenesis factor sFlt-1 from Median = 0.45 pg/ml in control groups to Median = 0.63 pg/ml (*p* < 0.001), whereas inserts of titanised meshes covered with fibroblasts under ceramic beads had no influence on the sFlt-1(Median = 0.37 pg/ml vs. Median = 0.45 pg/ml) expression of fibroblasts in comparison to controls but showed lower sFlt-1 expression (*p* < 0.001) in comparison to polypropylene meshes

Cell cultures of polypropylene mesh covered with fibroblasts under ceramic beads presented high significantly increased concentration of **sFlt-1** from median = 0.45 pg/ml in control groups to median = 0.63 pg/ml (*p* < 0.001). Whereas inserts of titanised meshes covered with fibroblasts under ceramic beads had no influence on the sFlt-1 expression of fibroblasts compared with controls (median = 0.37 pg/ml vs. median = 0.45 pg/ml) (Fig. 4).

**Fig. 5:**
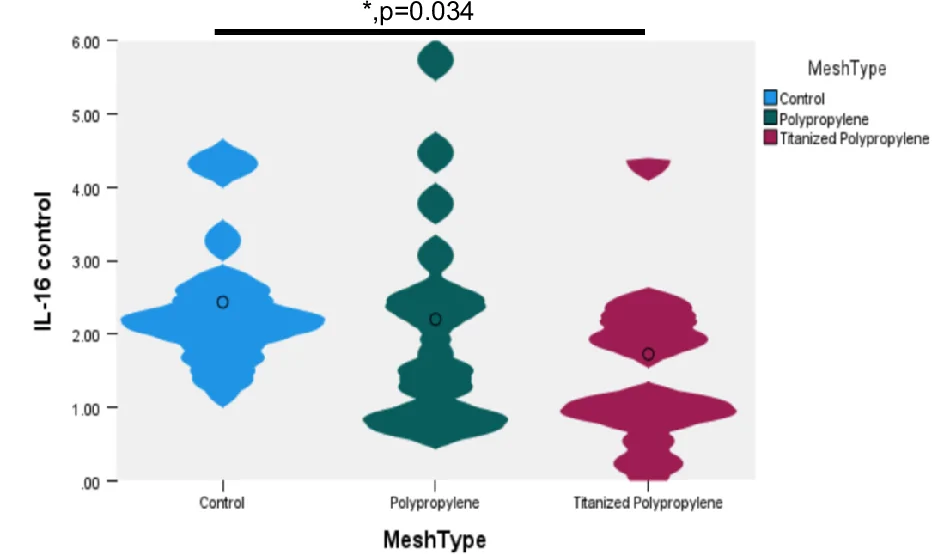
Cytokine IL-16 concentration was downregulated from Median = 2.19 pg/ml to Median = 1.82 pg/ml (*p* = 0.034) in cell cultures of titanised meshes covered with fibroblasts under ceramic beads. The cell cultures of polypropylene mesh covered with fibroblasts under ceramic beads presented no effect on the expression of cytokine IL-16 (Median = 1.93 pg/ml vs. Median = 2.19 pg/ml) in comparison to controls in 16 measurements (8 independent experiments measured in two samples)

**IL-16** concentration was modestly but statistically significant downregulated from median = 2.19 pg/ml in controls, to median = 1.82 pg/ml in cell cultures with titanised meshes covered with fibroblasts under ceramic beads (*p* = 0.034). Cell cultures of polypropylene mesh covered with fibroblasts under ceramic beads showed no effect on cytokine IL-16 expression compared to the control group (median = 1.93 pg/ml vs. median = 2.19 pg/ml) (Fig. 5).

**Fig. 6:**
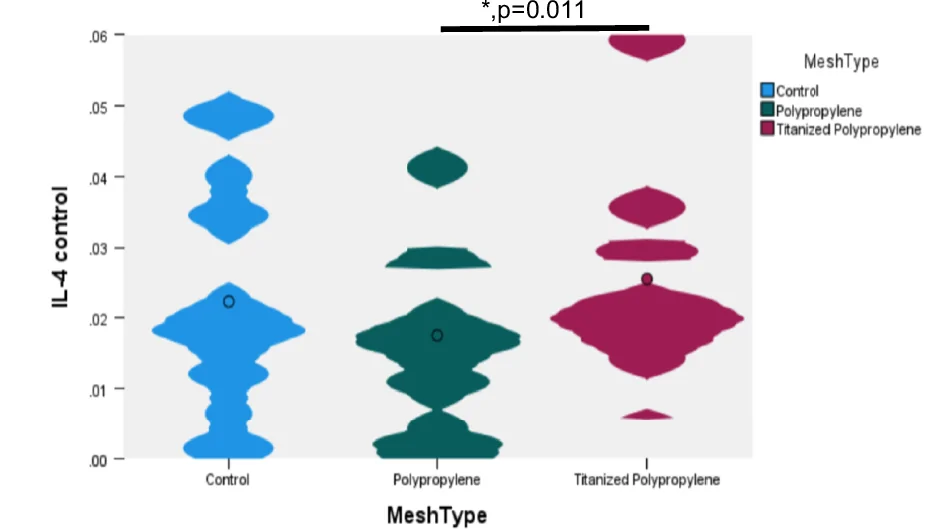
Inserts of polypropylene meshes covered with fibroblasts under ceramic beads downregulate the expression of cytokine IL-4(Median = 0.0171 pg/ml) compared with inserts of titanised meshes covered with fibroblasts under ceramic beads(Median = 0.0222 pg/ml) (*p* = 0.011), whereas cell cultures of titanised meshes covered with fibroblasts under ceramic beads did not present an effect on the cytokine IL-4 fibroblasts production in comparison to controls in 16 measurements (8 independent experiments measured in two samples)

The expression of **cytokine IL-4** was modestly but statistically significant down-regulated from median = 0.0171 pg/ml in cell cultures with polypropylene meshes covered with fibroblasts under ceramic beads in comparison to median = 0.0222 pg/ml in cell cultures with titanised meshes covered with fibroblasts under the ceramic beads (*p* = 0.011) (Fig. 6).

## Discussion

In this study, we evaluated the expression and regulation of angiogenesis factors VEGF, VEGF-C, and sFlt-1 in inserts with different meshes. We identified significant increases of VEGF and VEGF-C vascular angiogenesis factors for both meshes, while cell cultures with pure polypropylene meshes demonstrated significant upregulation of sFlt-1.

Additionally, we also analyzed the expression of pro- and anti-inflammatory cytokines. Titanised meshes were significantly downregulated the concentration of pro-inflammatory cytokine IL-16, whereas polypropylene meshes had no effect on the IL-16 cytokine fibroblast expression. Polypropylene meshes significantly reduced the expression of the anti-inflammatory cytokine IL-4, while titanised meshes had no effect on IL-4 production by fibroblasts.

### Surface Modification Effects of Titanised Meshes

Titanised meshes offer the advantage of modifying the surface of the polypropylene scaffold [44]. Recent research demonstrates that TiO₂ nanostructures have excellent bioactivity, stimulating adhesion, proliferation of fibroblasts and enhancing cell attachment to implant surfaces [45]. TiO₂ coatings significantly change surface hydrophilicity, making them biologically active and improving cellular responses [41]. Studies show that different crystalline phases of TiO₂ (anatase and rutile) provide favourable templates for biomedical implants, with enhanced fibroblast viability, adhesion, and proliferation [42]. The biocompatibility of titanium alloys used as implant material is markedly enhanced by TiO₂ modifications, which promote cellular adhesion and viability while reducing inflammatory responses[42].

### Angiogenic Response Differences

Taking together, our results on angiogenesis factors suggest that pure polypropylene meshes cancel or diminish the vascularization induction by upregulating sFlt-1, whereas titanised meshes show only stimulation of vascular angiogenesis factors. Vascular endothelial growth factors (VEGF) not only drive pro-angiogenic processes but also exert mitogenic and anti-apoptotic effects on endothelial cells, enhancing vascular permeability, promoting cellular migration, and supporting other key cellular functions [46,47].

sFLT-1, conversely, is recognized as an early marker for preeclampsia in pregnancy and contributes to endothelial dysfunction [48–52]. Additionally, sFlt-1 serves as negative prognostic biomarker in patients with acute coronary syndrome [53]. Importantly, sFlt-1 functions as a potent antagonist of VEGF bioactivity through direct reversible sequestration of VEGF ligands and dominant-negative heterodimerization with surface VEGF receptor monomers [54]. Research demonstrates that soluble VEGF receptors can inhibit angiogenic effects, and while VEGF function is essential for optimal wound angiogenesis, adenovirus-mediated gene transfer of soluble VEGF receptors significantly decreases wound angiogenesis without grossly delaying wound closure [55]. These findings indicate that the upregulation of sFlt-1 observed with polypropylene meshes may adversely affect the vascularization required for optimal tissue integration without necessarily compromising fundamental wound-healing processes.

In tissue repair contexts, elevated sFlt-1 levels are associated with impaired neovascularisation. For instance, aged ischaemic muscle exhibited reduced expression of pro-angiogenic factors alongside elevated sFlt-1 levels, with increased infiltration of inflammatory cells and heightened matrix metalloproteinase activity. Immunofluorescence analysis revealed that Flt-1 co-localised with macrophages in aged ischaemic muscles, thereby contributing to endothelial dysfunction [56,57].

### Inflammatory Cytokine Modulation

Pro-inflammatory IL-16 and anti-inflammatory IL-4 cytokine expressions were analysed in this study after co-cultivation with different mesh materials. Pro-inflammatory cytokine IL-16 likely plays a key role in postoperative complications such as erosion, infection, chronic pain and implant rejection [58–60]. A recent study by Fava et al demonstrated that IL-16 could play a pivotal role in driving inflammation by recruiting immune cells, including neutrophils and monocytes and facilitating crescent formation [61].

Regarding macrophages, Wen et al found that IL-16 enhances anti-tumour immune responses by establishing a Th1 cell-macrophage cross talk through reprogramming glutamine metabolism in mice [62]. Additionally, IL-16 regulates macrophage polarization as a target gene of mir-145-3p [63]. Finally, a research group found that IL-16 aggravates dextran sulphate sodium-induced mouse inflammatory bowel disease by promoting M1 polarization of macrophages [64]. Although nothing is known in literature about IL-16 and polypropylene meshes, a recent publication showed that Interleukin-16 rs4072111 polymorphism is associated with the risk of peri-Implantitis in the Chinese population [65].

Degranulation of neutrophils or monocytes can directly damage the endothelium and contribute to extracellular matrix remodelling [61]. In individuals with hidradenitis suppurativa, a chronic inflammatory condition marked by painful, deep-seated nodules, abscesses, and draining tunnels in skin areas such as the axilla, inguinal region, anogenital area, or inframammary folds, IL-16 levels were also found to be elevated [66]. Proinflammatory cytokine IL-16 is already recognized for its involvement in chronic inflammatory diseases. Allergic individuals have higher IL-16 amounts in their nasal secretions as compared to non-allergic individuals [67]. Therefore, upregulation of IL-16 as we observed in co-cultures of fibroblasts with polypropylene meshes can be associated with acute, chronic and allergic immune responses.

Simultaneously, anti-inflammatory cytokine IL-4 plays a central role in modulating immune responses by orchestrating diverse functions such as immune regulation, antibody synthesis, and promotion of tissue repair processes [68]. IL-4 is integral to tissue repair through two main pathways: first, it suppresses initial inflammatory responses to injury, and second, it regulates extracellular matrix modifications and rebuilding for effective repair [69]. However, the role of IL-4 in wound healing is complex and context-dependent.

Recent research reveals that IL-4 can both promote and impair wound healing depending on the circumstances. While IL-4 stimulates extracellular matrix synthesis and promotes collagen production [70], studies show that IL-4 can impair wound healing potential by repressing fibronectin expression in keratinocytes [71]. Fibronectin is a critical component of the extracellular matrix expressed at high levels in wound sites and is essential for wound repair through mechanisms including growth factor binding stability and cell survival. In experimental models, the topical application of IL-4 to wounds resulted in rapid enhancement of granulated tissue formation, but chronic IL-4 stimulation can lead to delayed re-epithelialisation [71].

Regarding macrophages and gynaecological problems, IL-4 induces M2 macrophage polarisation and can inhibit fibrosis in endometrial stromal cells [72]. IL-4 is also expressed on M2-polarized macrophages [73],and IL-4-treated human macrophages promote epithelial wound recovery and suppress inflammatory responses [74].

A recent study on IL-4 coated polypropylene mesh and its in vivo transplantation into living mice showed that IL-4 diminished the formation of fibrotic capsule surrounding the implant and improved tissue integration [75]. In addition, macrophage polarisation was influenced in an implant protective manner, clearly showing the positive effect of IL-4 in mesh tolerance [75]. Impaired regulation of Type 2 immune responses during wound healing can lead to fibrosis and scarring, with IL-4—a hallmark of Type 2 immune cells—playing a critical role in modulating this process [68,76,77]. The pleiotropic cytokine IL-4 can activate connective tissue cells and promote accumulation of extracellular matrix components. Therefore, downregulation of IL-4 in polypropylene meshes covered with fibroblasts could lead to impaired tissue repair processes and/or increased inflammatory responses, contrasting with the beneficial effects observed in titanised meshes.

In murine models, IL-4 signalling affects tissue repair in a context-dependent manner. While IL-4 can enhance granulation tissue formation when applied topically to experimental wounds (Salmon-Ehr, Implication of interleukin-4 in wound healing, 2000) [70], it can also suppress neutrophil spreading and migration by counteracting granulocyte colony-stimulating factor signaling pathways [78]. The context-dependent effects of IL-4 suggest that its downregulation by polypropylene meshes may have varying consequences depending on the healing environment and timing of mesh integration.

### Clinical Relevance and Previous Findings

In a recent study, we investigated the relationship between the in vivo and in vitro results of macrophage polarisation and immune checkpoint expression regarding different mesh material [17]. In particular, a massive influx of macrophages, accompanied by specific macrophage polarization, was observed in the non-titanised mesh surrounding patients in whom the mesh had been removed following complications related to a POP surgery.

As differential immune responses were observed only when mesh materials were covered with fibroblasts, this study focused on evaluating the effects of fibroblast (NHDF) coverage on the various mesh types. Consistent with our initial findings, polypropylene meshes elicited a more pronounced proinflammatory and antiangiogenic response, including increased sFlt-1 expression in NHDF cultures, thereby corroborating and extending the results of our previous study [17]. Additionally, these results may be of interest for further in vitro but also in vivo studies [79–82].

The experiments conducted in this study contribute to a better understanding of immune responses following mesh implantation and provides insights into the biological interactions between host tissue and different mesh materials. These findings may help inform future research and support evidence-based decision-making regarding mesh selection for POP treatment. However, determining the most appropriate material requires consideration of multiple clinical factors, including patient characteristics, surgical technique, and long-term outcomes, rather than relying on material properties alone. The latter point represents the major limitation of this study.

## Conclusion

Our in vitro findings appear to suggest that titanised polypropylene meshes could offer certain advantages over non‑titanised polypropylene meshes in pelvic organ prolapse surgery. In particular, titanised meshes were associated with (1) lower expression of the proinflammatory cytokine IL-16, which may be linked to a reduced risk of chronic inflammation, infection, or implant-related complications; (2) preservation of anti-inflammatory IL-4 levels, which could support tissue integration and wound healing; and (3) a more proangiogenic profile, without the sFlt-1 upregulation observed with pure polypropylene meshes, potentially facilitating improved vascularization and long-term implant integration.

Further validation in multicellular systems and clinically relevant in vivo models would further strengthen the results of our hypothesis-generating observations that require confirmation in more complex biological models.

## Data Availability

The raw data are available from the corresponding author upon request.

## References

[1] Collins, S., & Lewicky-Gaupp, C. (2022). Pelvic organ prolapse. *Gastroenterology Clinics of North America*, *51*(1), 177–193. 35135661 10.1016/j.gtc.2021.10.011

[2] Wu, J. M., Vaughan, C. P., Goode, P. S., Redden, D. T., Burgio, K. L., Richter, H. E., & Markland, A. D. (2014). Prevalence and trends of symptomatic pelvic floor disorders in U.S. women. *Obstetrics and Gynecology*, *123*(1), 141–148. 24463674 10.1097/AOG.0000000000000057 PMC3970401

[3] Ramalingam, K., & Monga, A. (2015). Obesity and pelvic floor dysfunction. *Best Practice and Research. Clinical Obstetrics and Gynaecology*, *29*(4), 541–547. 25805440 10.1016/j.bpobgyn.2015.02.002

[4] de Sam Lazaro, S., Nardos, R., & Caughey, A. B. (2016). Obesity and pelvic floor dysfunction: Battling the bulge. *Obstetrical & Gynecological Survey*, *71*(2), 114–125. 26894803 10.1097/OGX.0000000000000274

[5] Lince, S. L., van Kempen, L. C., Vierhout, M. E., & Kluivers, K. B. (2012). A systematic review of clinical studies on hereditary factors in pelvic organ prolapse. *International Urogynecology Journal*, *23*(10), 1327–1336. 22422218 10.1007/s00192-012-1704-4 PMC3448053

[6] Tinelli, A., Malvasi, A., Rahimi, S., Negro, R., Vergara, D., Martignago, R., Pellegrino, M., & Cavallotti, C. (2010). Age-related pelvic floor modifications and prolapse risk factors in postmenopausal women. *Menopause*, *17*(1), 204–212. 19629013 10.1097/gme.0b013e3181b0c2ae

[7] Jackson, S. R., Avery, N. C., Tarlton, J. F., Eckford, S. D., Abrams, P., & Bailey, A. J. (1996). Changes in metabolism of collagen in genitourinary prolapse. *Lancet*, *347*(9016), 1658–1661. 8642960 10.1016/s0140-6736(96)91489-0

[8] Patel, D. A., Xu, X., Thomason, A. D., Ransom, S. B., Ivy, J. S., & DeLancey, J. O. (2006). Childbirth and pelvic floor dysfunction: An epidemiologic approach to the assessment of prevention opportunities at delivery. *American Journal of Obstetrics and Gynecology*, *195*(1), 23–28. 16579934 10.1016/j.ajog.2006.01.042 PMC1486798

[9] Weintraub, A. Y., Glinter, H., & Marcus-Braun, N. (2020). Narrative review of the epidemiology, diagnosis and pathophysiology of pelvic organ prolapse. *International braz j urol : official journal of the Brazilian Society of Urology*, *46*(1), 5–14. 31851453 10.1590/S1677-5538.IBJU.2018.0581 PMC6968909

[10] Bø, K. (2012). Pelvic floor muscle training in treatment of female stress urinary incontinence, pelvic organ prolapse and sexual dysfunction. *World Journal of Urology*, *30*(4), 437–443. 21984473 10.1007/s00345-011-0779-8

[11] Griebling, T. L. (2016). Vaginal pessaries for treatment of pelvic organ prolapse in elderly women. *Current Opinion in Urology*, *26*(2), 201–206. 26765048 10.1097/MOU.0000000000000266

[12] Hagen, S., Glazener, C., McClurg, D., Macarthur, C., Elders, A., Herbison, P., Wilson, D., Toozs-Hobson, P., Hemming, C., Hay-Smith, J., Collins, M., Dickson, S., & Logan, J. (2017). Pelvic floor muscle training for secondary prevention of pelvic organ prolapse (PREVPROL): A multicentre randomised controlled trial. *Lancet*, *389*(10067), 393–402. 28010994 10.1016/S0140-6736(16)32109-2

[13] Hagen, S., & Stark, D. (2011). Conservative prevention and management of pelvic organ prolapse in women. *Cochrane Database of Systematic Reviews*, *2011*(12), CD003882. 22161382 10.1002/14651858.CD003882.pub4 PMC12621084

[14] Wiegersma, M., Panman, C. M. C. R., Hesselink, L. C., Malmberg, A. G. A., Berger, M. Y., Kollen, B. J., & Dekker, J. H. (2019). Predictors of success for pelvic floor muscle training in pelvic organ prolapse. *Physical Therapy*, *99*(1), 109–117. 30329105 10.1093/ptj/pzy114

[15] Viereck, V., Bader, W., Lobodasch, K., Pauli, F., Bentler, R., & Kolbl, H. (2016). Guideline-based strategies in the surgical treatment of female urinary incontinence: The new gold standard is almost the same as the old one. *Geburtshilfe Und Frauenheilkunde*, *76*(8), 865–868. 27570251 10.1055/s-0042-107079 PMC4999329

[16] Murphy, A. M., Clark, C. B., Denisenko, A. A., D’Amico, M. J., & Vasavada, S. P. (2021). Surgical management of vaginal prolapse: current surgical concepts. *The Canadian journal of urology*, *28*(S2), 22–26. 34453425

[17] Protsepko, O., Voisard, P., Kuhn, C., Maccagno, A., Dannecker, C., Jeschke, U., Pauli, F., & Garrido, F. (2023). Induction of a different immune response in non-titanized compared to titanized polypropylene meshes. *Acta biomaterialia*, *169*, 363–371. 37579913 10.1016/j.actbio.2023.08.018

[18] Xiao, Y., Zuo, X., Li, H., Zhao, Y., & Wang, X. (2023). Impact of titanium-coated polypropylene mesh on functional outcome and quality of life after inguinal hernia repair. *Heliyon*, *9*(7), e17691. 37455954 10.1016/j.heliyon.2023.e17691 PMC10345250

[19] Köckerling, F., & Schug-Pass, C. (2014). What do we know about titanized polypropylene meshes? An evidence-based review of the literature. *Hernia*, *18*(4), 445–457. 24253381 10.1007/s10029-013-1187-3 PMC4113678

[20] Campagna, G., Pedone Anchora, L., Panico, G., Caramazza, D., Arcieri, M., Cervigni, M., Scambia, G., & Ercoli, A. (2020). Titanized polypropylene mesh in laparoscopic sacral colpopexy. *International Urogynecology Journal*, *31*(4), 763–768. 31807800 10.1007/s00192-019-04146-x

[21] Cadenbach-Blome, T., Grebe, M., Mengel, M., Pauli, F., Greser, A., & Funfgeld, C. (2019). Significant improvement in quality of life, positive effect on sexuality, lasting reconstructive result and low rate of complications following cystocele correction using a lightweight, large-pore, titanised polypropylene mesh: Final results of a national, multicentre observational study. *Geburtshilfe Und Frauenheilkunde*, *79*(9), 959–968. 31523096 10.1055/a-0984-6614 PMC6739206

[22] Guo, T., Hu, X., Du, Z., Wang, X., Lang, J., Liu, J., Xu, H., & Sun, Z. (2024). Modification of transvaginal polypropylene mesh with co-axis electrospun nanofibrous membrane to alleviate complications following surgical implantation. *Journal of Nanobiotechnology*, *22*(1), 598. 39363196 10.1186/s12951-024-02872-z PMC11447934

[23] Ai, F. F., Mao, M., Zhang, Y., Kang, J., & Zhu, L. (2020). Experimental study of a new original mesh developed for pelvic floor reconstructive surgery. *International Urogynecology Journal*, *31*(1), 79–89. 30997545 10.1007/s00192-019-03947-4

[24] Lu, C., Zhou, J., Kong, Q., Wang, L., Ni, W., & Xiao, Z. (2025). New rat model mimicking sacrocolpopexy for POP treatment and biomaterials testing via unilateral presacral suspension. *International Urogynecology Journal*, *36*(2), 421–429. 39777526 10.1007/s00192-024-06019-4 PMC11850470

[25] Kim, Y. H., Panda, A. K., & Shevach, E. M. (2025). Treg control of CD80/CD86 expression mediates immune system homeostasis. *European Journal of Immunology*, *55*(5), e202551771. 40346769 10.1002/eji.202551771 PMC12064877

[26] Wang, Q., Schmoeckel, E., Kost, B. P., Kuhn, C., Vattai, A., Vilsmaier, T., Mahner, S., Mayr, D., Jeschke, U., & Heidegger, H. H. (2019). Higher CCL22+ cell infiltration is associated with poor prognosis in cervical cancer patients. *Cancers*, *11*(12), 2004. 31842422 10.3390/cancers11122004 PMC6966573

[27] Mannewitz, M., Kolben, T., Perleberg, C., Meister, S., Hahn, L., Mitter, S., Schmoeckel, E., Mahner, S., Corradini, S., Trillsch, F., Kessler, M., Jeschke, U., & Beyer, S. (2024). CCL22 as an independent prognostic factor in endometrial cancer patients. *Translational Oncology*, *50*, 102116. 39232378 10.1016/j.tranon.2024.102116 PMC11404215

[28] Boutilier, A. J., & Elsawa, S. F. (2021). Macrophage polarization states in the tumor microenvironment. *International Journal of Molecular Sciences*, *22*(13), 6995. 34209703 10.3390/ijms22136995 PMC8268869

[29] Wang, L. X., Zhang, S. X., Wu, H. J., Rong, X. L., & Guo, J. (2019). M2b macrophage polarization and its roles in diseases. *Journal of Leukocyte Biology*, *106*(2), 345–358. 30576000 10.1002/JLB.3RU1018-378RR PMC7379745

[30] Toyokawa, H., Nakao, A., Bailey, R. J., Nalesnik, M. A., Kaizu, T., Lemoine, J. L., Ikeda, A., Tomiyama, K., Papworth, G. D., Huang, L., Demetris, A. J., Starzl, T. E., & Murase, N. (2008). Relative contribution of direct and indirect allorecognition in developing tolerance after liver transplantation. *Liver Transplantation*, *14*(3), 346–357. 18306376 10.1002/lt.21378 PMC3022430

[31] McGuigan, A. J., Coleman, H. G., McCain, R. S., Kelly, P. J., Johnston, D. I., Taylor, M. A., & Turkington, R. C. (2021). Immune cell infiltrates as prognostic biomarkers in pancreatic ductal adenocarcinoma: A systematic review and meta-analysis. *The Journal of Pathology: Clinical Research*, *7*(2), 99–112. 33481339 10.1002/cjp2.192 PMC7869931

[32] Takeuchi, S., Fujiyama, S., Nagafuji, M., Mayumi, M., Saito, M., Obata-Yasuoka, M., Hamada, H., Miyazono, Y., & Takada, H. (2025). Nucleated red blood cells secrete haptoglobin to induce immunosuppressive function in monocytes. *Journal of Immunology Research*, *2025*, 8085784. 40017804 10.1155/jimr/8085784 PMC11867727

[33] Komohara, Y., Kurotaki, D., Tsukamoto, H., Miyasato, Y., Yano, H., Pan, C., Yamamoto, Y., & Fujiwara, Y. (2023). Involvement of protumor macrophages in breast cancer progression and characterization of macrophage phenotypes. *Cancer science*, *114*(6), 2220–2229. 36748310 10.1111/cas.15751 PMC10236637

[34] Gianchecchi, E., & Fierabracci, A. (2018). Inhibitory receptors and pathways of lymphocytes: The role of PD-1 in treg development and their involvement in autoimmunity onset and cancer progression. *Frontiers in immunology*, *9*, 2374. 30386337 10.3389/fimmu.2018.02374 PMC6199356

[35] Tang, Q., Chen, Y., Li, X., Long, S., Shi, Y., Yu, Y., Wu, W., Han, L., & Wang, S. (2022). The role of PD-1/PD-L1 and application of immune-checkpoint inhibitors in human cancers. *Frontiers in immunology*, *13*, 964442. 36177034 10.3389/fimmu.2022.964442 PMC9513184

[36] Li, W., Wu, F., Zhao, S., Shi, P., Wang, S., & Cui, D. (2022). Correlation between PD-1/PD-L1 expression and polarization in tumor-associated macrophages: A key player in tumor immunotherapy. *Cytokine and Growth Factor Reviews*, *67*, 49–57. 35871139 10.1016/j.cytogfr.2022.07.004

[37] Qian, H. Y., Chen, L., Zhang, X. M., Qiu, L., Wang, F., Feng, T., Shan, J., Yuan, X., & Chen, X. L. (2025). Molecular insight of nanosized Ba-Hao herbal ointment in accelerating chronic wound healing. *Nanoscale Advances*, *7*(11), 3406–3413. 40270832 10.1039/d4na01075b PMC12012563

[38] Stygar, D., Pogorzelska, A., Chelmecka, E., Skrzep-Poloczek, B., Bazanow, B., Gebarowski, T., Jochem, J., & Henych, J. (2021). Graphene oxide normal (GO + Mn(2+)) and ultrapure: Short-term impact on selected antioxidant stress markers and cytokines in NHDF and A549 cell lines. *Antioxidants (Basel)*, *10*(5), 765. 34065001 10.3390/antiox10050765 PMC8151183

[39] Plencner, M., Prosecká, E., Rampichová, M., East, B., Buzgo, M., Vysloužilová, L., Hoch, J., & Amler, E. (2015). Significant improvement of biocompatibility of polypropylene mesh for incisional hernia repair by using poly-ε-caprolactone nanofibers functionalized with thrombocyte-rich solution. *International journal of nanomedicine*, *10*, 2635–2646. 25878497 10.2147/IJN.S77816 PMC4388102

[40] Cheng, H., Zhang, Y., Zhang, B., Cheng, J., Wang, W., Tang, X., Teng, P., & Li, Y. (2017). Biocompatibility of polypropylene mesh scaffold with adipose-derived stem cells. *Experimental and Therapeutic Medicine*, *13*(6), 2922–2926. 28587361 10.3892/etm.2017.4338 PMC5450661

[41] Riivari, S., Narva, E., Kangasniemi, I., Willberg, J., & Narhi, T. (2022). Epithelial cell attachment and adhesion protein expression on novel in sol TiO(2) coated zirconia and titanium alloy surfaces. *Journal of biomedical materials research. Part B, Applied biomaterials*, *110*(11), 2533–2541. 35730701 10.1002/jbm.b.35111 PMC9543659

[42] Jafari, S., Mahyad, B., Hashemzadeh, H., Janfaza, S., Gholikhani, T., & Tayebi, L. (2020). Biomedical applications of TiO(2) nanostructures: Recent advances. *International journal of nanomedicine*, *15*, 3447–3470. 32523343 10.2147/IJN.S249441 PMC7234979

[43] U-PLEX biomarker group 1 (human) multiplex assays (access date 07/15/2025)

[44] Areid, N., Riivari, S., Abushahba, F., Shahramian, K., & Narhi, T. (2023). Influence of surface characteristics of TiO(2) coatings on the response of gingival cells: A systematic review of in vitro studies. *Materials (Basel)*, *16*(6), 2533. 36984413 10.3390/ma16062533 PMC10056999

[45] Dias-Netipanyj, M. F., Sopchenski, L., Gradowski, T., Elifio-Esposito, S., Popat, K. C., & Soares, P. (2020). Crystallinity of TiO(2) nanotubes and its effects on fibroblast viability, adhesion, and proliferation. *Journal of Materials Science. Materials in Medicine*, *31*(11), 94. 33128627 10.1007/s10856-020-06431-4

[46] Melincovici, C. S., Boşca, A. B., Şuşman, S., Mărginean, M., Mihu, C., Istrate, M., Moldovan, I. M., Roman, A. L., & Mihu, C. M. (2018). Vascular endothelial growth factor (VEGF) - key factor in normal and pathological angiogenesis. *Romanian Journal of Morphology and Embryology*, *59*(2), 455–467. 30173249

[47] Zhou, Y., Zhu, X., Cui, H., Shi, J., Yuan, G., Shi, S., & Hu, Y. (2021). The role of the VEGF family in coronary heart disease. *Frontiers in Cardiovascular Medicine*, *8*, 738325. 34504884 10.3389/fcvm.2021.738325 PMC8421775

[48] Schaarschmidt, W., Rana, S., & Stepan, H. (2012). PP052. The course of sFlt-1 and PLGF reflects different progression pattern in early- versus late-onset preeclampsia and HELLP syndrome. *Pregnancy Hypertension*, *2*(3), 269. 26105375 10.1016/j.preghy.2012.04.163

[49] Stepan, H., Hund, M., Gencay, M., Denk, B., Dinkel, C., Kaminski, W. E., Wieloch, P., Semus, B., Meloth, T., Dröge, L. A., & Verlohren, S. (2016). A comparison of the diagnostic utility of the sFlt-1/PlGF ratio versus PlGF alone for the detection of preeclampsia/HELLP syndrome. *Hypertension in Pregnancy*, *35*(3), 295–305. 27028698 10.3109/10641955.2016.1141214 PMC5309866

[50] Suzuki, H., Nagayama, S., Hirashima, C., Takahashi, K., Takahashi, H., Ogoyama, M., Nagayama, M., Shirasuna, K., Matsubara, S., & Ohkuchi, A. (2018). Markedly higher sFlt-1/PlGF ratio in a woman with acute fatty liver of pregnancy compared with HELLP syndrome. *The Journal of Obstetrics and Gynaecology Research*, *45*, 96–103. 30141235 10.1111/jog.13786

[51] Zhao, Y., Zheng, Y., Liu, X., Luo, Q., Wu, D., Liu, X., & Zou, L. (2018). Inhibiting trophoblast PAR-1 overexpression suppresses sFlt-1-induced anti-angiogenesis and abnormal vascular remodeling: a possible therapeutic approach for preeclampsia. *Molecular Human Reproduction*, *24*(3), 158–169. 29325127 10.1093/molehr/gax068

[52] Zhou, Q., Qiao, F. Y., Zhao, C., & Liu, H. Y. (2011). Hypoxic trophoblast-derived sFlt-1 may contribute to endothelial dysfunction: implication for the mechanism of trophoblast-endothelial dysfunction in preeclampsia. *Cell Biology International*, *35*(1), 61–66. 20828367 10.1042/CBI20100020

[53] Marks, E. C. A., Wilkinson, T. M., Frampton, C. M., Skelton, L., Pilbrow, A. P., Yandle, T. G., Pemberton, C. J., Doughty, R. N., Whalley, G. A., Ellis, C. J., Troughton, R. W., Owen, M. C., Pattinson, N. R., Cameron, V. A., Richards, A. M., Gieseg, S. P., & Palmer, B. R. (2018). Plasma levels of soluble VEGF receptor isoforms, circulating pterins and VEGF system SNPs as prognostic biomarkers in patients with acute coronary syndromes. *BMC Cardiovascular Disorders*, *18*(1), 169. 30111293 10.1186/s12872-018-0894-1 PMC6094571

[54] Wu, F. T., Stefanini, M. O., Mac Gabhann, F., Kontos, C. D., Annex, B. H., & Popel, A. S. (2010). VEGF and soluble VEGF receptor-1 (sFlt-1) distributions in peripheral arterial disease: an in silico model. *American Journal of Physiology. Heart and Circulatory Physiology*, *298*(6), H2174–H2191. 20382861 10.1152/ajpheart.00365.2009 PMC2886617

[55] Jacobi, J., Tam, B. Y., Sundram, U., von Degenfeld, G., Blau, H. M., Kuo, C. J., & Cooke, J. P. (2004). Discordant effects of a soluble VEGF receptor on wound healing and angiogenesis. *Gene Therapy*, *11*(3), 302–309. 14737090 10.1038/sj.gt.3302162

[56] Zhai, Y., Liu, Y., Qi, Y., Long, X., Gao, J., Yao, X., Chen, Y., Wang, X., Lu, S., & Zhao, Z. (2020). The soluble VEGF receptor sFlt-1 contributes to endothelial dysfunction in IgA nephropathy. *PLoS ONE*, *15*(8), e0234492. 32790760 10.1371/journal.pone.0234492 PMC7425938

[57] Zhao, G., Cheng, X. W., Piao, L., Hu, L., Lei, Y., Yang, G., Inoue, A., Ogasawara, S., Wu, H., Hao, C. N., Okumura, K., & Kuzuya, M. (2017). The soluble VEGF receptor sFlt-1 contributes to impaired neovascularization in aged mice. *Aging and Disease*, *8*(3), 287–300. 28580185 10.14336/AD.2016.0920 PMC5440109

[58] Zhang, J., Zhao, W., Zhou, Y., Xi, S., Xu, X., Du, X., Zheng, X., Hu, W., Sun, R., Tian, Z., Fu, B., & Wei, H. (2024). Pyroptotic T cell-derived active IL-16 has a driving function in ovarian endometriosis development. *Cell Reports Medicine*, *5*(3), 101476. 38508138 10.1016/j.xcrm.2024.101476 PMC10983113

[59] Zhu, X., Liu, S., Tian, L., Li, X., Yao, R., Zhao, Y., Gao, Z., Liu, X. R., Liu, X. Q., Huo, F. Q., & Liang, L. (2024). Spinal interleukin-16 mediates inflammatory pain via promoting glial activation. *International Immunopharmacology*, *127*, 111411. 38113689 10.1016/j.intimp.2023.111411

[60] González-Rodríguez, S., Sordo-Bahamonde, C., Álvarez-Artime, A., Baamonde, A., & Menéndez, L. (2024). Hyperalgesic effect evoked by il-16 and its participation in inflammatory hypernociception in mice. *Journal of neuroimmune pharmacology: the official journal of the Society on NeuroImmune Pharmacology*, *19*(1), 44. 39152360 10.1007/s11481-024-10145-7 PMC11329551

[61] Fava, A., Buyon, J., Magder, L., Hodgin, J., Rosenberg, A., Demeke, D. S., Rao, D. A., Arazi, A., Celia, A. I., Putterman, C., Anolik, J. H., Barnas, J., Dall'Era, M., Wofsy, D., Furie, R., Kamen, D., Kalunian, K., James, J. A., Guthridge, J., & Petri, M. (2024). Urine proteomic signatures of histological class, activity, chronicity, and treatment response in lupus nephritis. *JCI Insight*, *9*(2), e172569. 38258904 10.1172/jci.insight.172569 PMC10906224

[62] Wen, Z., Liu, T., Xu, X., Acharya, N., Shen, Z., Lu, Y., Xu, J., Guo, K., Shen, S., Zhao, Y., Wang, P., Li, S., Chen, W., Li, H., Ding, Y., Shang, M., Guo, H., Hou, Y., Cui, B., & Xiao, P. (2025). Interleukin-16 enhances anti-tumor immune responses by establishing a Th1 cell-macrophage crosstalk through reprogramming glutamine metabolism in mice. *Nature Communications*, *16*(1), 2362. 40064918 10.1038/s41467-025-57603-1 PMC11893787

[63] Huang, Y., Du, K. L., Guo, P. Y., Zhao, R. M., Wang, B., Zhao, X. L., & Zhang, C. Q. (2019). IL-16 regulates macrophage polarization as a target gene of mir-145-3p. *Molecular Immunology*, *107*, 1–9. 30634164 10.1016/j.molimm.2018.12.027

[64] Zhu, Y., Dai, J., Yao, X., Dong, G., Zhang, H., Yan, F., Li, C., Li, X., Xiong, H., & Si, C. (2018). [IL-16 aggravates dextran sulfate sodium (DSS)-induced mouse inflammatory bowel disease by promoting M1 polarization of macrophages]. *Xi bao yu fen zi mian yi xue za zhi = Chinese journal of cellular and molecular immunology*, *34*(8), 695–701. 30384867

[65] Chen, Z., & Chen, G. (2021). Interleukin-16 rs4072111 polymorphism is associated with the risk of peri-implantitis in the chinese population. *Pharmacogenomics and Personalized Medicine*, *14*, 1629–1635. 34938097 10.2147/PGPM.S336857 PMC8686223

[66] Jorgensen, A. R., Thomsen, S. F., Karmisholt, K. E., & Ring, H. C. (2020). Clinical, microbiological, immunological and imaging characteristics of tunnels and fistulas in hidradenitis suppurativa and Crohn’s disease. *Experimental Dermatology*, *29*(2), 118–123. 31519056 10.1111/exd.14036

[67] Sudharson, S., Eckl-Dorna, J., Meshcheryakova, A., Basílio, J., Derdak, S., Kalic, T., Lengger, N., Schweitzer, N., Mechtcheriakova, D., Breiteneder, H., & Hafner, C. (2024). Transcriptomic profiles of the nasal mucosa following birch pollen provocation differ between birch pollen-allergic and non-allergic individuals. *Allergy*, *80*, 1757–1769. 39717878 10.1111/all.16448 PMC12186594

[68] Bernstein, Z. J., Shenoy, A., Chen, A., Heller, N. M., & Spangler, J. B. (2023). Engineering the IL-4/IL-13 axis for targeted immune modulation. *Immunological Reviews*, *320*(1), 29–57. 37283511 10.1111/imr.13230

[69] Allen, J. E. (2023). IL-4 and IL-13: Regulators and effectors of wound repair. *Annual Review of Immunology*, *41*, 229–254. 36737597 10.1146/annurev-immunol-101921-041206

[70] Salmon-Ehr, V., Ramont, L., Godeau, G., Birembaut, P., Guenounou, M., Bernard, P., & Maquart, F. X. (2000). Implication of interleukin-4 in wound healing. *Laboratory Investigation*, *80*(8), 1337–1343. 10950124 10.1038/labinvest.3780141

[71] Serezani, A. P. M., Bozdogan, G., Sehra, S., Walsh, D., Krishnamurthy, P., Sierra Potchanant, E. A., Nalepa, G., Goenka, S., Turner, M. J., Spandau, D. F., & Kaplan, M. H. (2017). IL-4 impairs wound healing potential in the skin by repressing fibronectin expression. *The Journal of Allergy and Clinical Immunology*, *139*(1), 142–151.e145. 27554818 10.1016/j.jaci.2016.07.012 PMC5222746

[72] Feng, D., Li, Y., Zheng, H., Wang, Y., Deng, J., Liu, T., Liao, W., & Shen, F. (2024). IL-4-induced M2 macrophages inhibit fibrosis of endometrial stromal cells. *Reproductive biology*, *24*(2), 100852. 38354656 10.1016/j.repbio.2023.100852

[73] Mercnik, M. H., Schliefsteiner, C., Fluhr, H., & Wadsack, C. (2023). Placental macrophages present distinct polarization pattern and effector functions depending on clinical onset of preeclampsia. *Frontiers in Immunology 2022*, *13*, 1095879. 10.3389/fimmu.2022.1095879 PMC9878680 36713449

[74] Jayme, T. S., Leung, G., Wang, A., Workentine, M. L., Rajeev, S., Shute, A., Callejas, B. E., Mancini, N., Beck, P. L., Panaccione, R., & McKay, D. M. (2020). Human interleukin-4-treated regulatory macrophages promote epithelial wound healing and reduce colitis in a mouse model. *Science Advances*, *6*(23), eaba4376. 32548267 10.1126/sciadv.aba4376 PMC7274799

[75] Hachim, D., LoPresti, S. T., Yates, C. C., & Brown, B. N. (2017). Shifts in macrophage phenotype at the biomaterial interface via IL-4 eluting coatings are associated with improved implant integration. *Biomaterials*, *112*, 95–107. 27760399 10.1016/j.biomaterials.2016.10.019 PMC5121003

[76] Gieseck, R. L., Wilson, M. S., & Wynn, T. A. (2018). Type 2 immunity in tissue repair and fibrosis. *Nature reviews. Immunology*, *18*(1), 62–76. 28853443 10.1038/nri.2017.90

[77] Wynn, T. A. (2015). Type 2 cytokines: mechanisms and therapeutic strategies. *Nature reviews. Immunology*, *15*(5), 271–282. 25882242 10.1038/nri3831

[78] Woytschak, J., Keller, N., Krieg, C., Impellizzieri, D., Thompson, R. W., Wynn, T. A., Zinkernagel, A. S., & Boyman, O. (2016). Type 2 Interleukin-4 Receptor Signaling in Neutrophils Antagonizes Their Expansion and Migration during Infection and Inflammation. *Immunity*, *45*(1), 172–184. 27438770 10.1016/j.immuni.2016.06.025

[79] Pelekanou, V., Villarroel-Espindola, F., Schalper, K. A., Pusztai, L., & Rimm, D. L. (2018). CD68, CD163, and matrix metalloproteinase 9 (MMP-9) co-localization in breast tumor microenvironment predicts survival differently in ER-positive and -negative cancers. *Breast cancer research : BCR*, *20*(1), 154. 30558648 10.1186/s13058-018-1076-x PMC6298021

[80] Ramalingam, S., Shantha, S., Srinivasan, C. P., Priyathersini, N., Muralitharan, S., Sudhakar, U., Thamizhchelvan, H., & Parvathi, V. D. (2024). Expression of mTOR, CD163, alpha-SMA, FOXp3 as survival predictors and its significance in patients with oral squamous cell carcinoma. *BMC Oral Health*, *24*(1), 1487. 39695576 10.1186/s12903-024-05245-y PMC11653707

[81] Shabo, I., Olsson, H., Sun, X. F., & Svanvik, J. (2009). Expression of the macrophage antigen CD163 in rectal cancer cells is associated with early local recurrence and reduced survival time. *International Journal of Cancer*, *125*(8), 1826–1831. 19582880 10.1002/ijc.24506

[82] Shabo, I., Stal, O., Olsson, H., Dore, S., & Svanvik, J. (2008). Breast cancer expression of CD163, a macrophage scavenger receptor, is related to early distant recurrence and reduced patient survival. *International Journal of Cancer*, *123*(4), 780–786. 18506688 10.1002/ijc.23527

